# Grapevine rust fungus *Neophysopella tropicalis* deploys protein effectors with plant immunity-suppressing activity during sequential stages of pathogenesis

**DOI:** 10.1007/s00299-026-03980-w

**Published:** 2026-10-21

**Authors:** Thiago Maia, João Vitor Ferro, Gustavo Husein, Renato G. H. Bombardelli, Claudia B. Monteiro-Vitorello

**Affiliations:** 1 Center for Plant Science Innovation Department of Plant Pathology and, University of Nebraska-Lincoln, Lincoln, NE United States; 2 Departamento de Genética, Universidade de São Paulo (USP), Escola Superior de Agricultura Luiz de Queiroz (ESALQ), Piracicaba, SP Brasil

**Keywords:** Biotrophic fungi, Pathogenesis, Effectoromics, RNA-seq, *Vitis labrusca*, WGA-FITC microscopy, *Pseudomonas fluorescens* EtHAn, AvrB

## Abstract

Germination of urediniospores marks the onset of infection by *Neophysopella tropicalis*, the causal agent of Asian grapevine leaf rust, yet the molecular mechanisms enabling this obligate biotrophic fungus to establish infection remain largely unknown. We combined transcriptomics, histological analyses, and functional assays to identify and characterize candidate effector proteins deployed during the earliest stages of pathogenesis. RNA sequencing of in vitro-germinated urediniospores identified 303 genes encoding predicted secreted proteins bearing hallmarks of fungal effectors, grouped into 142 sequence families and revealing a remarkably diverse repertoire of effector candidates. Fifteen highly expressed *N. tropicalis* effector candidate (*NtEC*) genes, representing thirteen distinct sequence families, were selected for expression profiling by reverse transcription quantitative PCR across infection stages. Chitin-targeted fluorescence microscopy provided a developmental framework for interpreting expression across germination, penetration, and early colonization. Although identified during germination, seven NtECs accumulated transcripts predominantly during host penetration and early biotrophic colonization, suggesting specialized roles in infection establishment. Using the Type III Secretion System of *Pseudomonas fluorescens* EtHAn, we demonstrated that three NtECs (NtEC-05, NtEC-09, and NtEC-10) strongly suppressed AvrB-triggered hypersensitive cell death in *Nicotiana benthamiana*, while three others (NtEC-11, NtEC-12, and NtEC-13) exhibited moderate immune-suppressing activity. Together, these findings provide the first comprehensive characterization of the *N. tropicalis* effector repertoire, demonstrating that early infection involves coordinated, sequential deployment of evolutionarily diverse effectors associated with plant immune suppression. This work establishes an integrated framework for discovery and functional prioritization of rust fungus effectors and provides a valuable molecular resource for future studies on grapevine–rust interactions.

**Supplementary Information:**

The online version contains supplementary material available at https://doi.org/10.1007/s00299-026-03980-w.

## Introduction

Grapevine (*Vitis* spp.) ranks among the world’s most important fruit crops and, in recent years, has gained increasing prominence in Brazil (de Oliveira et al. 2020). For fresh fruit consumption, the American *Vitis labrusca* cultivars predominate in Brazilian vineyards*,* mainly due to their vegetative traits well adapted to the country’s environmental conditions (Santos et al. 2020a; da Silva et al. 2022). However, maintaining this crop requires significant investment in integrated disease management combining cultural and chemical control (Gramaje et al. 2018). The problem is especially acute for *V. labrusca*, which is vulnerable to a wide range of filamentous plant pathogens (Primiano et al. 2017; Santos et al. 2020b). Among these, *Neophysopella tropicalis*, the causal agent of Asian grapevine leaf rust (AGLR), stands out as one of the most destructive threats to the crop (Rasera et al. 2023). AGLR was initially attributed to species within the genus *Phakopsora*, but has since been reassigned to the genus *Neophysopella* based on morphological and phylogenetic analyses (Ji et al. 2019).

A survey of Brazilian *Neophysopella* isolates associated with AGLR revealed that all 50 monouredinial cultures from the south-central region were identified as *N. tropicalis*, whereas six isolates from the northeast were classified as *Neophysopella meliosmae-myrianthae* (Santos et al. 2020a). Therefore, *N. tropicalis* is the predominant causal agent of AGLR in Brazil and the sole species found in the southern and southeastern regions, which together account for approximately 75% of the nation’s grape production. Despite its high prevalence and destructive potential, little is known about the molecular mechanisms underlying the virulence of *N*. *tropicalis*. In particular, the repertoire of effector proteins deployed during the earliest stages of infection and their role in suppressing plant immunity remain completely unexplored.

A hallmark of rust fungi is their large secretome, encompassing hundreds of predicted genes encoding secreted proteins, including effectors relevant to establishing infection and manipulation of the host immunity and physiology (Figueroa et al. 2021). Genomic and transcriptomic studies have shown that rust fungal effectors are frequently organized into highly diverse and lineage-specific repertoires that evolve rapidly under the selective pressure imposed by host immune systems (Saunders et al. 2012; Lorrain et al. 2019). Characterizing these repertoires is therefore fundamental for understanding pathogen virulence mechanisms and identifying molecular targets for developing durable disease resistance. In *N. tropicalis*, urediniospores germination represents the initial and critical step in pathogenesis, marking the onset of infection. Understanding the molecular events occurring during this stage is thus essential to elucidate how filamentous pathogens penetrate and colonize their hosts (Shen et al. 2017; He et al. 2020).

To investigate these early infection processes, germinating urediniospores from obligate biotrophic pathogens generated in vitro provide a valuable biological source for RNA sequencing (RNA-seq) studies. This strategy allows isolation of pathogen RNA free from plant biomass. Moreover, candidate effector genes identified from complementary DNA (cDNA) libraries constructed from germinating urediniospores frequently exhibit transcript accumulation in planta between 24 and 72 h post-inoculation (Maia et al. 2017), confirming that germlings serve as a valuable biological source for identifying effectors induced during plant infection.

In this study, we developed a robust RNA-seq strategy using germinating urediniospores of *N. tropicalis* to identify candidate effector genes expressed at early infection stages and functionally characterize their role in suppressing plant immunity. Our objectives were to: (i) identify genes encoding candidate effectors expressed during urediniospores germination, (ii) analyze the diversity of the candidate effector repertoire through sequence-based clustering, (iii) profile candidate effector expression throughout infection stages, (iv) associate effector expression with distinct stages of pathogenesis, and (v) establish a transient expression system for *N*. *tropicalis* secreted proteins in planta using the Type III Secretion System of *Pseudomonas fluorescens* EtHAn. This comprehensive investigation fills a critical knowledge gap in *N. tropicalis* biology, providing novel insights into the molecular mechanisms underlying its pathogenicity and supporting future efforts to develop grapevine cultivars resistant to this destructive disease. Notably, no candidate effector sequences from *N. tropicalis* are currently available in GenBank or other public databases, highlighting the urgent need for genomic and transcriptomic resources to uncover the virulence mechanisms of this economically important rust fungus.

## Materials and methods

### Urediniospores germination

Urediniospores from the single-pustule isolate Aglr64 of *N*. *tropicalis*, originally collected in Jales, São Paulo State, Brazil, kindly provided by the group of Professor Lilian Amorim (ESALQ-USP) and previously molecularly characterized by Santos et al. (2020a), were inoculated onto the abaxial surface of fully expanded, healthy leaves of *V. labrusca* cv. Niagara Rosada to promote multiplication under controlled growth chamber conditions, following Rasera et al. (2023). Ten days after inoculation, leaves exhibiting a high density of sporulating pustules were harvested, and approximately two grams of fresh urediniospores were collected by gently brushing the abaxial surface of infected leaves. The urediniospores were then washed with sterile distilled water containing 0.05% Tween 80 (Sigma-Aldrich), evenly distributed onto polystyrene Petri dishes with a thin film of sterile water, and incubated in darkness at 22 °C. Germination was confirmed by light microscopy after 16 h of incubation. The resulting *N. tropicalis* germlings were collected, flash-frozen in liquid nitrogen, and stored at –80 °C until total RNA extraction.

### RNA preparation and RNA-seq library for sequencing

Total RNA was extracted from germinated urediniospores using RNeasy Plant Mini Kit (Qiagen), according to the manufacturer’s instructions. Any genomic DNA contamination was eliminated from the extracted RNA using RNase-free DNase I, following the manufacturer’s protocol (Sigma-Aldrich). RNA quality was assessed by a 2100 Bioanalyzer RNA Nano Chip (Agilent). Library construction was performed using the TruSeq™ Stranded mRNA Library Preparation Kit (Illumina) and then paired-end sequenced on a NextSeq 500 platform (Illumina) at the Functional Genomics Center, ESALQ, University of São Paulo (Piracicaba, São Paulo, Brazil).

### De novo transcriptome assembly and candidate effector protein predictions

FastQC v0.11.5 was used to check read quality (Andrew 2010). We then trimmed and quality-filtered reads using Cutadapt v1.18 (Martin 2011) to remove adapters and keep only reads longer than 50 bp, with no “N” bases and an average Phred quality score higher than 30. For transcriptome assembly, we used Trinity v2.14 (Grabherr et al. 2011) with default parameters. The input used was the filtered FastQ files from the biological triplicate. The completeness of the de novo transcriptome assembly was assessed using BUSCO v5.6.0 (Manni et al. 2021) against the fungi_odb10 lineage dataset to estimate the recovery of conserved single-copy fungal orthologs. As this study focused on the identification of candidate effectors, we ran SignalP v6.0 (Teufel et al. 2022), TMHMM v2.0 (Krogh et al. 2001), and EffectorP v3.0 (Sperschneider and Dodds 2022) (*P* > 0.8) to predict the candidate effectors from *N*. *tropicalis*. We mapped the reads against the de novo transcriptome assembly using Hisat2 v2.2.1 software (Kim et al. 2015) and generated a count table using FeatureCounts from the subread package v2.0.6 (Liao et al. 2014) to estimate the most expressed candidate effector genes in germinated urediniospores of *N*. *tropicalis*. Subsequently, the final set of predicted effectors was annotated using BlastP against the non-redundant National Center for Biotechnology Information (NCBI) protein database.

### Clustering analysis of the predicted effector repertoire

To characterize the diversity of the *N. tropicalis* effector repertoire, the predicted N-terminal signal peptides were removed from all candidate effectors, and the resulting mature protein sequences were clustered into putative sequence families using CD-HIT v4.8.1 based on global amino acid sequence identity (Huang et al. 2010). A 40% sequence identity threshold was applied to define cluster representatives and assign homologous sequences to the same family.

### Selection and validation of the candidate effector genes using RT-PCR

The fifteen most highly expressed candidate effector genes with complete open reading frames (ORFs) were selected to validate the bioinformatic predictions by reverse transcription polymerase chain reaction (RT-PCR). For cDNA synthesis, 1.0 µg of total RNA extracted from germinated urediniospores of the pathogen was used as input, with the Oligo(dT)12–18 primer and the SuperScript First-Strand Synthesis System (Thermo Scientific) RT-PCR kit. Gene-specific primers were designed to amplify the ORF sequences, excluding the signal peptide, using the synthesized cDNA as a template. PCR amplification was performed using the Phusion High-Fidelity PCR Master Mix (Thermo Scientific). The resulting amplicons were subsequently used for cloning and downstream functional analyses.

### Expression analysis of *N*. *tropicalis* candidate effector genes using RT-qPCR

For reverse transcription quantitative PCR (RT-qPCR) assays, inoculations were performed using a suspension of 1 × 10^5^ urediniospores mL⁻^1^, following Rasera et al. (2023). Total RNA was extracted from rust-infected grapevine leaves of *V. labrusca* cv. Niagara Rosada collected at 24, 48, and 72 h post-inoculation (hpi) and at 7 days post-inoculation (dpi), following a CTAB-based protocol (Iandolino et al. 2004). For each time point, three biological replicates, each consisting of independently infected leaves, were collected. Additionally, RNA from dormant and germinated urediniospores was isolated using the RNeasy Plant Mini Kit (Qiagen). Following DNase treatment, 1 µg of RNA from each sample was used for cDNA synthesis with the SuperScript First-Strand Synthesis System for RT-PCR (Invitrogen), using the Oligo(dT)12–18 primer in a final reaction volume of 20 µl. The quality and efficiency of cDNA synthesis were evaluated using endogenous genes from both the pathogen and the host (Supplementary Fig. S1).

Relative gene expression was calculated using the 2^−ΔCt^ method (Livak and Schmittgen 2001), based on the average Ct values from three biological replicates and two technical replicates per time point. RT-qPCR reactions were carried out using the GoTaq RT-qPCR Kit (Promega) on a 7300 PCR System (Applied Biosystems), with the following cycling conditions: initial denaturation at 95 °C for 2 min, followed by 40 cycles of 95 °C for 15 s and 60 °C for 60 s. Amplification specificity was confirmed by melting curve analysis, performed from 60 to 95 °C with a temperature increment of 1 °C every 30 s.

### Microscopy analyses of *N*. *tropicalis* infection stages

Fluorescence microscopy was performed using a Leica DM4000 microscope. Fungal hyphae were visualized by staining with 20 μg/mL wheat germ agglutinin conjugated to fluorescein isothiocyanate (WGA-FITC; Sigma-Aldrich), following the protocol described by Marrafon-Silva et al. (2024). FITC fluorescence was excited at 488 nm, and emission was detected within 500–550 nm range. To observe *N. tropicalis* infection structures, rust-inoculated grapevine leaves were collected at 24, 48, and 72 hpi, as well as at 5, 7, and 9 dpi.

### Cloning the *N*. *tropicalis* effector candidate genes into pEDV6 vector

*NtEC* sequences were cloned into the pEDV6 vector using the Gateway® LR Clonase™ II Enzyme Mix kit (Invitrogen), following the manufacturer’s instructions. The in-frame fusion and sequence integrity of all pEDV6::*NtEC* constructs were verified by sequencing. *Escherichia coli* DH5α was used for propagation of all pEDV6::*NtEC* constructs. The pEDV6 plasmids harboring *NtECs* were transferred from *E. coli* DH5α to *P. fluorescens* EtHAn by standard triparental mating, using *E. coli* HB101 (pRK2013) as the helper strain (Maia et al. 2022). Empty pEDV6 vectors were maintained in *E. coli* DB3.1. Transconjugated *P. fluorescens* EtHAn cells carrying the pEDV6::*NtEC* constructs were selected on solid Luria–Bertani (LB) medium supplemented with chloramphenicol (30 μg mL⁻^1^) and gentamicin (25 μg mL⁻^1^).

### Functional analysis of *N*. *tropicalis* effector candidate genes using the pEDV6 delivery system

To assess whether the NtECs can suppress plant immunity response, we employed an AvrB-induced cell death suppression assay (Maia et al. 2022). Suspensions of *P. fluorescens* EtHAn carrying pVSP6::*AvrB* (Innes et al. 1993) and *P. fluorescens* EtHAn carrying pEDV6::*NtECs* were mixed in a 1:1 ratio to a final concentration of 2 × 10⁷ colony-forming units (CFU) ml⁻^1^ and co-infiltrated into leaves of 4–5-week-old *Nicotiana benthamiana* plants using a needleless syringe. Co-infiltrations of *P. fluorescens* EtHAn (pVSP6::*AvrB*) with *P. fluorescens* EtHAn (pEDV6 empty vector) served as negative controls. Cell death suppression was considered to occur when an effector candidate reduced or prevented tissue collapse in at least 40% of the co-infiltrated leaves.

### Structural prediction of* N. tropicalis* effectors

Three-dimensional structures of NtECs with plant immune suppression activity (excluding signal peptides) were predicted using AlphaFold v3 (Abramson et al. 2024) using default settings, selecting the top-ranked of five models (model_0.cif). High-confidence structures, defined by a predicted TM-score (pTM > 0.5), were compared to known proteins via FoldSeek (van Kempen et al. 2024), aligned using US-align (Zhang et al. 2022), and visualized in PyMOL v3.1 (Schrödinger and DeLano 2020).

## Results

### Urediniospores germination and de novo transcriptome assembly

**Fig. 1:**
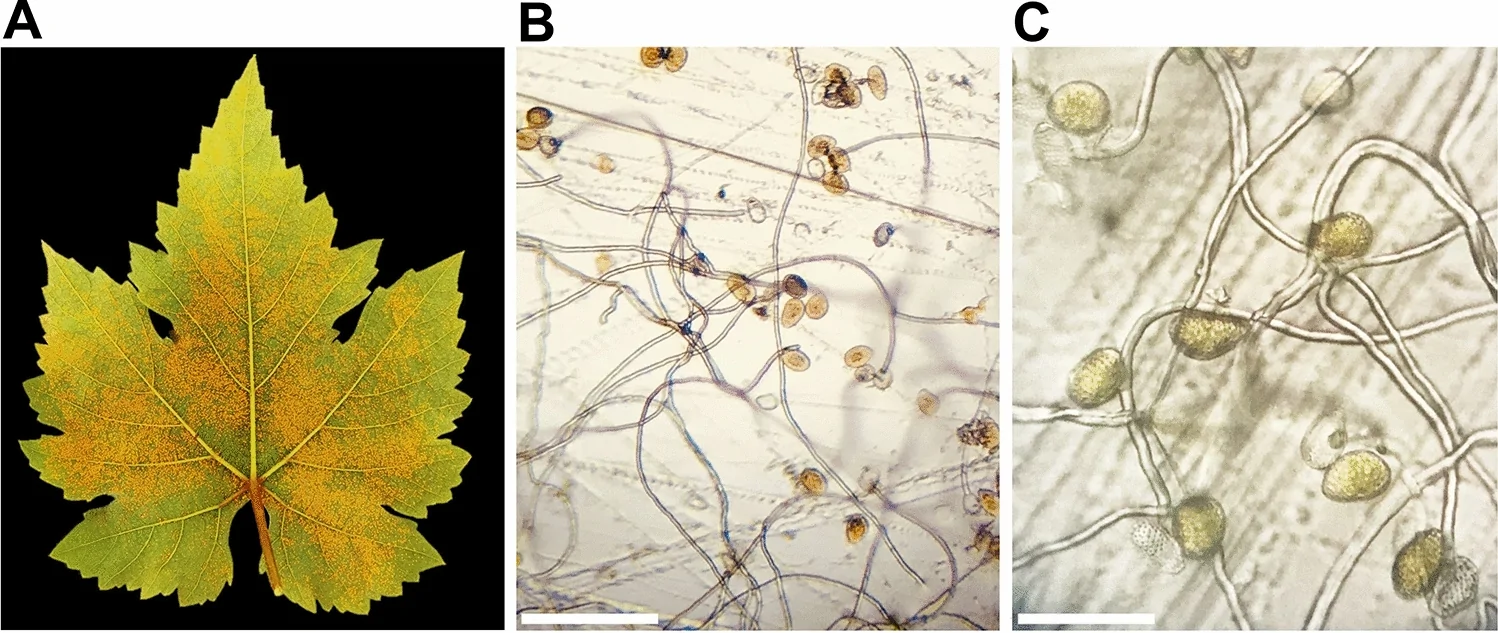
Asian grapevine rust fungus *Neophysopella tropicalis*. **A** Uredinial pustules of *N*. *tropicalis* isolate Aglr64 developing on the abaxial surface of *Vitis labrusca* cv. Niagara Rosada leaves. **B** Light microscopy image of germinated urediniospores and elongating germ tubes on polystyrene after 16 h of incubation at 22 °C. Scale bar = 120 µm. **C** Close-up view of germ tube emergence from urediniospores on the plate surface. Scale bar = 30 µm

Fresh urediniospores of *N*. *tropicalis* harvested from *V. labrusca* leaves (Fig. 1A) were washed and placed to germinate on polystyrene plates. After 16 h of incubation, 88% of the urediniospores had developed long germ tubes (Fig. 1B, C). Germinated urediniospores were then collected for total RNA extraction (Supplementary Fig. S2) and subsequent RNA-seq library construction.

High-throughput sequencing yielded over 90 million trimmed reads, which were assembled into contigs using the Trinity assembler. The final assembly consisted of 64,333 transcripts, of which 28,968 (45.03%) exceeded 1000 base pairs in length (Supplementary Table S1). BUSCO analysis revealed that 89.6% of the transcripts showed similarities with fungal sequences deposited in the fungi_odb10 database.

Among the assembled sequences, transcripts DN4595_c0_g1_i2 and DN303_c3_g2_i3 encoded full-length open reading frames (ORFs) with strong similarity to β-tubulin. DN4595_c0_g1_i2 (*NtβTub1*) shared 100% identity with a known *N. tropicalis* β-tubulin gene (GenBank accession UQJ88286.1), whereas DN303_c3_g2_i3 (*NtβTub2*) represents a putative novel β-tubulin gene not previously reported in this species, suggesting the existence of a second β-tubulin isoform.

To assess their conservation, we aligned the deduced amino acid sequences of *NtβTub1* and *NtβTub2* to their respective top BLASTP hits (Supplementary Fig. S3). In addition, two other contigs, DN600_c1_g1_i2 and DN600_c1_g1_i4, encoded full-length ORFs with high similarity to glyceraldehyde-3-phosphate dehydrogenase (*GAPDH*). These two sequences likely correspond to allelic variants of a single-copy gene, as they differ by only one nonsynonymous substitution (Supplementary Fig. S4), consistent with reports of single-copy *GAPDH* genes in other filamentous fungi (Lima et al. 2009). Currently, no *GAPDH* sequence from *N. tropicalis* is available in the NCBI GenBank database. These full-length *β-tubulin* and *GAPDH* sequences identified here served as endogenous reference genes for *N. tropicalis* to normalize the relative expression of candidate effector genes in forthcoming experiments.

### Clustering analysis of candidate effectors expressed during urediniospore germination

Among the 64,333 transcripts identified in the germinated urediniospore transcriptome of *N*. *tropicalis*, 6081 (9.45%) encoded predicted secreted proteins lacking transmembrane domains. Of these, 303 sequences were classified as candidate effectors using EffectorP (*P* > 0.8). To investigate the diversity and organization of the effector repertoire expressed during urediniospore germination, signal peptides were removed from the 303 predicted candidate effectors, and the mature protein sequences were clustered based on sequence similarity using CD-HIT with a 40% amino acid identity threshold. This analysis grouped the 303 candidate effectors into 142 putative sequence families (Supplementary Table S2).

**Fig. 2:**
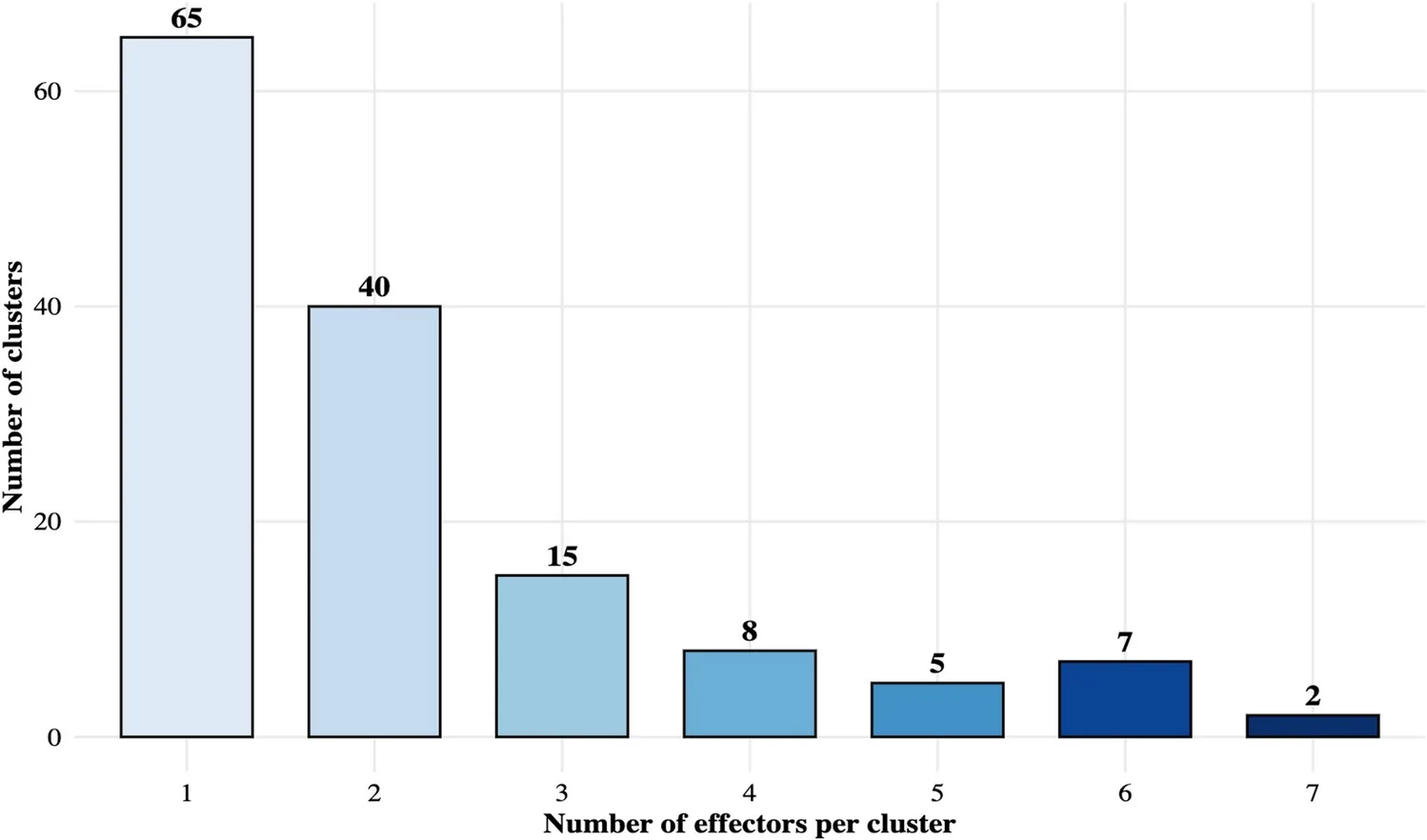
Clustering analysis of candidate effectors expressed during urediniospore germination of *Neophysopella tropicalis*. Predicted candidate effectors (*n* = 303) were grouped into families using CD-HIT following removal of signal peptides and applying a 40% amino acid identity threshold. The histogram shows the distribution of family sizes, with the x-axis indicating the number of candidate effectors per cluster and the y-axis indicating the number of clusters

Of these families, 65 (45.8%) were represented by a single member, whereas the remaining 77 contained between two and seven proteins (Fig. 2). The predominance of singleton and small multi-member families indicates limited expansion of individual effector lineages and reveals a highly diverse repertoire composed largely of sequence-divergent candidate effectors rather than members of large, expanded families.

### Characterization of the most highly expressed candidate effectors

We selected the 15 most highly expressed *N*. *tropicalis* effector candidate genes (*NtECs*) based on the number of reads mapped per transcript identified in the RNA-seq library (Table 1). Of the 15 candidate effectors selected for further characterization, 7 (*NtEC*-01, *NtEC*-07, *NtEC*-09, *NtEC*-11, *NtEC*-13, *NtEC*-14, and *NtEC*-15) encoded predicted proteins that showed no similarity to any proteins currently available in the NCBI non-redundant (nr) protein database based on BLASTP searches, suggesting that they may represent *N*. *tropicalis*-specific proteins lacking detectable homologs in current databases (Table 2). The predicted proteins encoded by *NtEC*-03, *NtEC*-05, *NtEC*-08, and *NtEC*-12 were exclusively homologous to proteins from rust fungi, indicating a potential specificity to the order Pucciniales. Three candidates (*NtEC*-02, *NtEC*-04, and *NtEC*-06) encoded predicted proteins that showed taxonomically restricted similarity to proteins of the Basidiomycota phylum. In contrast, the predicted NtEC-10 protein exhibited broader cross-phyla conservation, with significant similarity to proteins from both Basidiomycota and Ascomycota, pointing to a more evolutionarily conserved effector candidate (Table 2).

**Table 1:** Fifteen *Neophysopella tropicalis* effector candidate genes (*NtECs*) selected from the germinated urediniospores transcriptome library based on their high transcript abundance (read count per contig)

*NtEC*	Contig ID	ORF length	Mature ORF	No. of reads	Protein size (No. aa)	SignalP 6.0^*^
*NtEC-*01	DN7996_c0_g1_i12	480 bp	426 bp	22,927	160	0.981
*NtEC-*02	DN490_c0_g1_i5	498 bp	444 bp	14,308	166	0.9996
*NtEC-*03	DN4194_c0_g3_i1	471 bp	402 bp	11,488	157	0.9977
*NtEC-*04	DN62_c0_g1_i24	681 bp	624 bp	8101	227	0.9695
*NtEC-*05	DN470_c0_g1_i12	384 bp	324 bp	3505	128	0.9997
*NtEC-*06	DN62_c0_g1_i8	540 bp	480 bp	2305	180	0.8636
*NtEC-*07	DN2816_c0_g2_i23	531 bp	420 bp	1287	177	0.9941
*NtEC-*08	DN1789_c0_g1_i7	693 bp	639 bp	1098	231	0.9997
*NtEC-*09	DN8063_c0_g1_i13	474 bp	408 bp	1066	158	0.9998
*NtEC-*10	DN507_c0_g1_i2	378 bp	318 bp	996	126	0.9993
*NtEC-*11	DN2062_c0_g1_i5	486 bp	414 bp	968	162	0.9998
*NtEC-*12	DN7996_c0_g1_i17	480 bp	429 bp	869	160	0.9998
*NtEC-*13	DN3898_c0_g1_i2	306 bp	246 bp	761	102	0.9998
*NtEC-*14	DN1304_c0_g1_i6	360 bp	294 bp	743	120	0.9998
*NtEC-*15	DN7682_c0_g3_i1	519 bp	459 bp	715	173	0.9995

^*^Signal peptide predicted using SignalP 6.0 (Teufel et al. 2022)

**Table 2:** BLAST-based annotation of fifteen *Neophysopella tropicalis* effector candidates (NtECs) using the NCBI non-redundant (nr) database

*NtEC*	BLASTP (top hit)				
	description	Scientific name	Accession	E-value	Taxonomy
*NtEC-*01	No hit^*^	−	−	−	−
*NtEC-*02	Hypothetical protein	*Puccinia triticina*	OAV92395.1	5,00E−75	Basidiomycota
*NtEC-*03	Secreted protein	*Melampsora americana*	KAH9815948.1	4,00E−30	Pucciniales
*NtEC-*04	Hypothetical protein	*Puccinia graminis f.* sp.* tritici*	KAA1101121.1	4,00E−123	Basidiomycota
*NtEC-*05	Hypothetical protein	*Austropuccinia psidii*	MBW0508534.1	1,00E−15	Pucciniales
*NtEC-*06	Hypothetical protein	*Puccinia graminis* f. sp. *tritici*	KAA1101121.1	4,00E−63	Basidiomycota
*NtEC-*07	No hit^*^	−	−	−	−
*NtEC-*08	Hypothetical protein	*Austropuccinia psidii*	MBW0480326.1	2,00E−90	Pucciniales
*NtEC-*09	No hit^*^	−	−	−	−
*NtEC-*10	Hypothetical protein	*Cronartium quercuum*	KAG0143304.1	8,00E−41	Basidiomycota/Ascomycota
*NtEC-*11	No hit^*^	−	−	−	−
*NtEC-*12	Hypothetical protein	*Austropuccinia psidii*	MBW0501908.1	6,00E−09	Pucciniales
*NtEC-*13	No hit^*^	−	−	−	−
*NtEC-*14	No hit^*^	−	−	−	−
*NtEC-*15	No hit^*^	−	−	−	−

^*^No significant similarity found

**Fig. 3:**
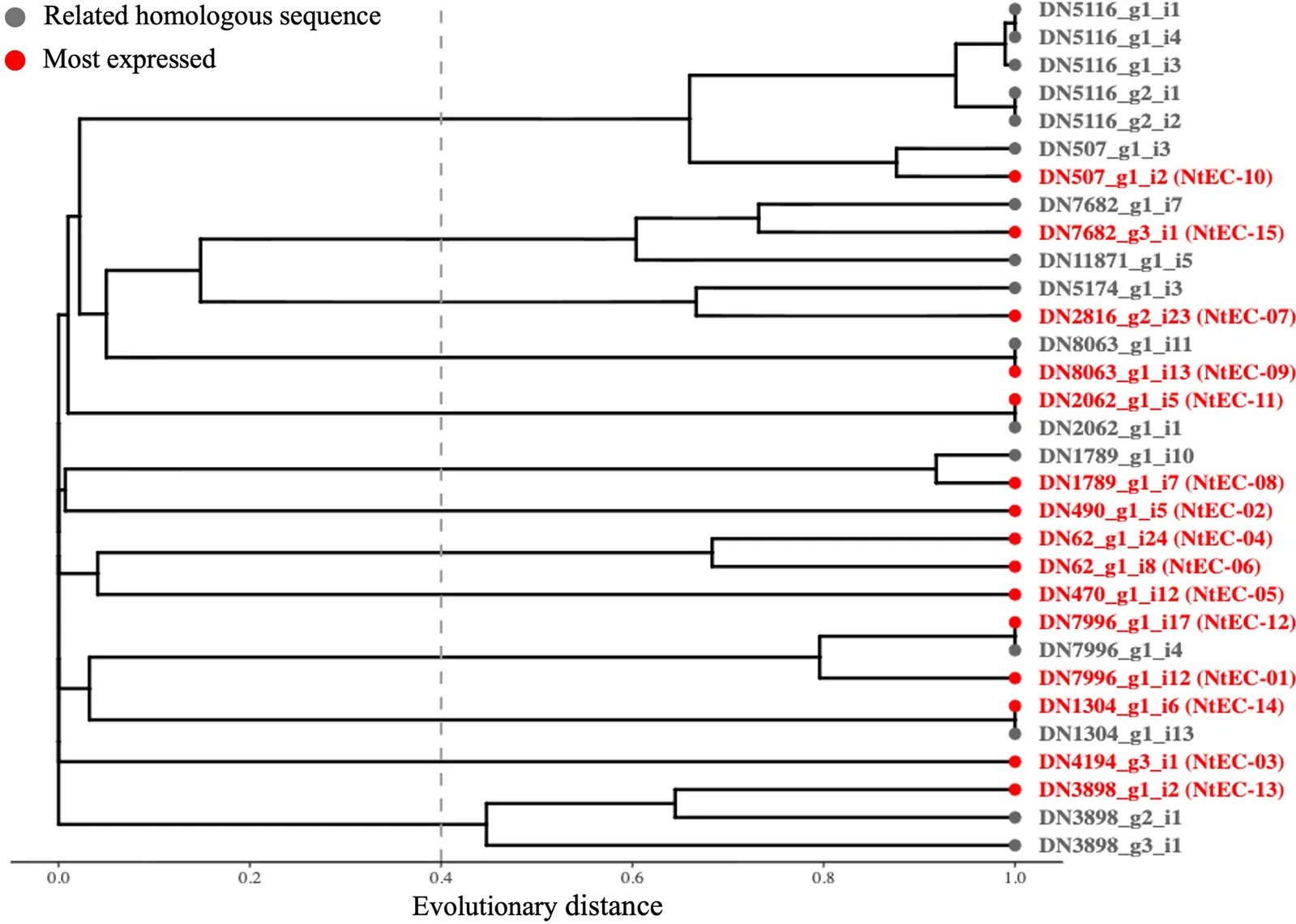
Sequence relationships among the fifteen most highly expressed candidate effectors of *Neophysopella tropicalis***.** Hierarchical clustering of the fifteen selected candidate effector (NtECs) and their homologous sequences identified from the germinated urediniospore transcriptome. Candidate effectors were assigned to the sequence families identified by CD-HIT using a 40% amino acid identity threshold. The selected NtECs (red labels) represent 13 distinct sequence families, whereas gray labels indicate homologous sequences or transcript variants within each family. The dashed vertical line denotes the 40% amino acid sequence identity threshold used to define homologous sequences included in the clustering analysis. Evolutionary distance (x-axis) represents 1 minus the fractional amino acid sequence identity between sequences. See Supplementary Table S3 for the complete list of homologous sequences associated with each NtEC family

To better understand the sequence relationships of the most highly expressed candidate effectors, the 15 NtECs were assigned to the sequence families identified by CD-HIT clustering and analyzed together with their homologous sequences using hierarchical clustering (Fig. 3; Supplementary Table S3). The selected 15 NtECs were distributed across 13 distinct sequence families, indicating that they represent a broad cross-section of the candidate effector repertoire rather than members of only a few expanded lineages. Only two pairs of highly expressed effectors, NtEC-01/NtEC-12 and NtEC-04/NtEC-06, belonged to the same sequence families, whereas the remaining 11 candidates each represented independent families. Several families, including those represented by NtEC-07, NtEC-10, NtEC-13, and NtEC-15, contained additional homologous sequences or transcript variants, while other NtECs formed isolated branches within the dendrogram, reflecting limited sequence similarity to other candidate effectors identified in the germination transcriptome. Collectively, these results demonstrate that the most highly expressed NtECs encompass a broad spectrum of sequence diversity, ranging from representatives of moderately sized effector families to highly differentiated candidates with few closely related homologs.

**Fig. 4:**
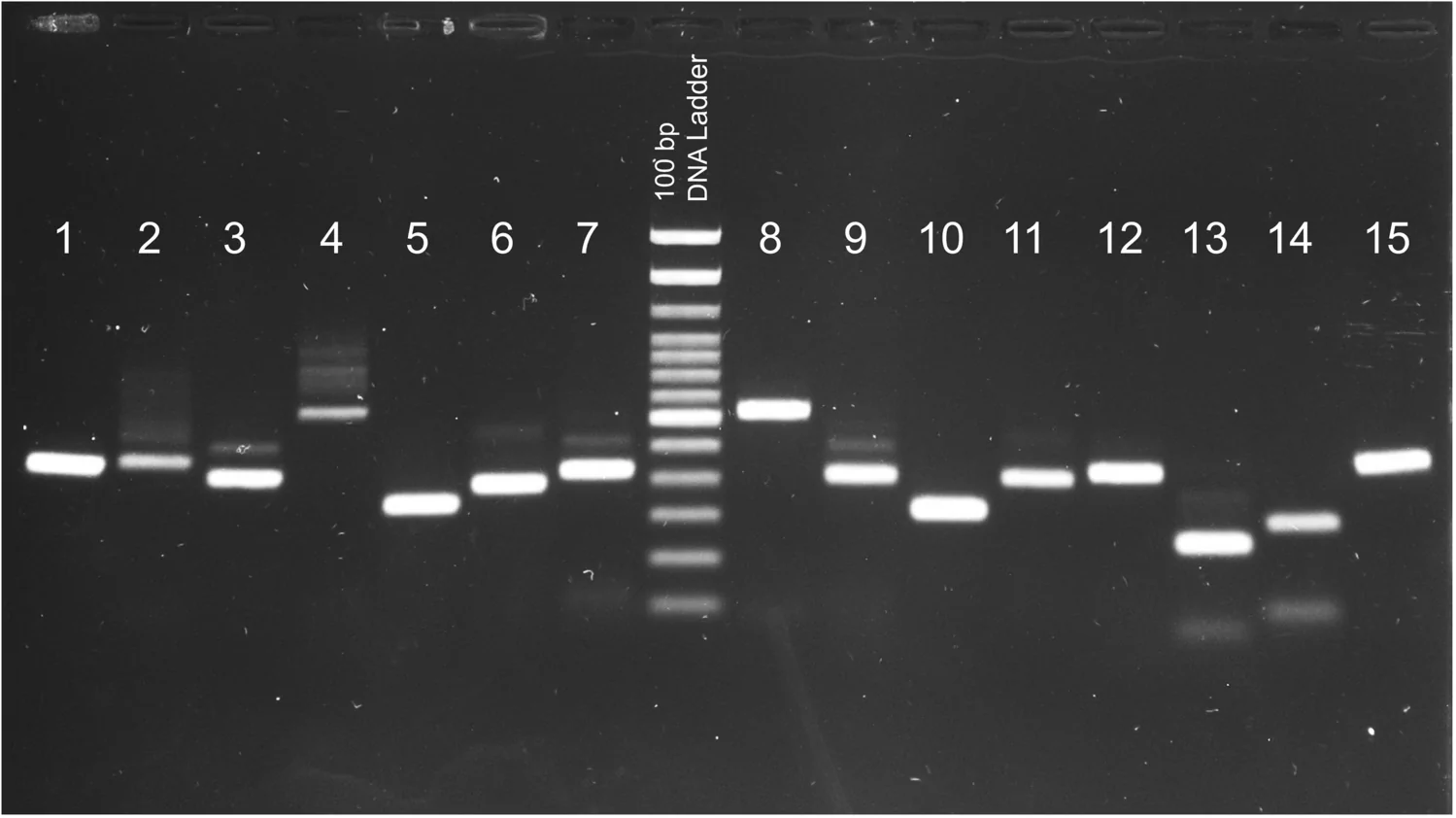
Amplification of fifteen *Neophysopella tropicalis* effector candidate genes (*NtECs*) from germinated urediniospores by reverse transcription polymerase chain reaction (RT-PCR). Agarose gel electrophoresis (1.2%) showing RT-PCR products amplified from cDNA synthesized using RNA extracted from germinated urediniospores. Each amplicon corresponds to the mature open reading frame (ORF) of the respective effector candidate gene, excluding the N-terminal signal peptide sequence; expected sizes are listed in Table 1. Lane assignments: 1: *NtEC-*01 (426 bp), 2: *NtEC-*02 (444 bp), 3: *NtEC-*03 (402 bp), 4: *NtEC-*04 (624 bp), 5: *NtEC-*05 (324 bp), 6: *NtEC-*06 (380 bp)*, 7: *NtEC-*07 (420 bp), 8: *NtEC-*08 (639 bp), 9: *NtEC-*09 (408 bp), 10: *NtEC-*10 (318 bp), 11: *NtEC-*11 (414 bp), 12: *NtEC-*12 (429 bp), 13: *NtEC-*13 (246 bp), 14: *NtEC-*14 (294 bp), 15: *NtEC-*15 (459 bp). Primer sequences are listed in Table S4. *NtEC-06 was the only amplicon whose observed size (380 bp) did not match the size predicted from the mature ORF (480 bp; see Table 1)

To experimentally validate the bioinformatic predictions and expression of the most highly expressed *N. tropicalis* candidate effectors, we conducted RT-PCR assays using cDNA synthesized from total RNA extracted from germinated urediniospores. Primers were designed to amplify the coding region of the mature protein, excluding the N-terminal signal peptide sequence predicted by SignalP 6.0. Fourteen of the fifteen *NtECs* produced amplicons of the expected size, consistent with the in silico predictions of mature ORFs (Fig. 4; Table 1). One exception was *NtEC-*06, which generated an amplicon of approximately 380 bp instead of the predicted 480 bp. This corresponds to an overall validation accuracy of 93.33% for our computational predictions. The fourteen successfully validated *NtECs* were further subjected to gene expression profiling during the pre-biotrophic and biotrophic stages of *N. tropicalis* development to investigate their potential roles in distinct stages of pathogenesis.

### Expression profiling of *N. tropicalis* candidate effectors during in vitro and in planta development

We analyzed transcript accumulation of *NtEC* genes at distinct stages of the grapevine rust fungus life cycle. To this end, cDNA was synthesized from RNA extracted from both dormant and germinated urediniospores, as well as from infected grapevine leaves collected at 24, 48, and 72 hpi, and 7 dpi. To verify the quality of the cDNA derived from these samples, RT-PCR was performed using primers targeting the fungal endogenous reference genes *NtβTub1* and *NtβTub2* (β-tubulin), *NtGAPDH* (glyceraldehyde-3-phosphate dehydrogenase), and *NtCytIII* (cytochrome c oxidase subunit III), along with the grapevine reference gene *VvActin7* (Supplementary Fig. S1). All 14 *NtECs* yielded a single amplification product in samples from urediniospores and/or germinated urediniospores. Some *NtECs* were also amplified in infected leaf tissues, indicating that a subset of these genes may be expressed during the biotrophic phase of infection (Supplementary Fig. S1).

**Fig. 5:**
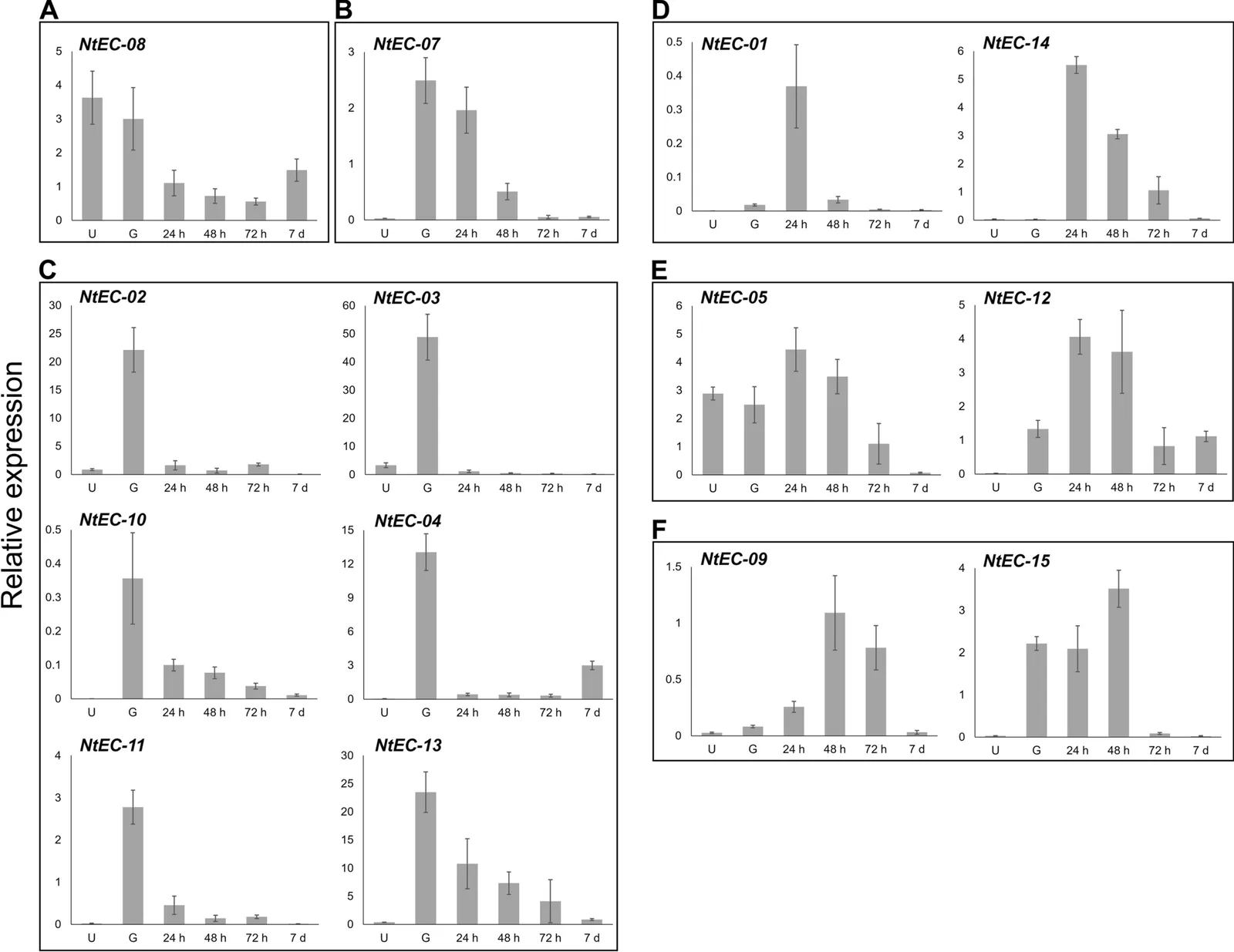
Expression profiles of selected *Neophysopella tropicalis* effector candidate genes (*NtECs*) identified from the germinated urediniospore transcriptome. Transcript accumulation of *NtECs* was assessed by real-time quantitative PCR (RT-qPCR) in samples obtained from dormant urediniospores (U), germinated urediniospores on polystyrene plates (G), and infected *Vitis labrusca* cv. Niagara Rosada leaves at 24, 48, and 72 h post-inoculation (hpi), and 7 days post-inoculation (dpi). Genes were grouped into panels **A–F** based on shared expression patterns: **A** *NtEC* with highest expression in dormant and germinated urediniospores; **B** *NtEC* with highest peaks in germinated urediniospores and at 24 hpi; **C** *NtECs* with peak expression exclusively in germinated urediniospores; **D** *NtECs* with highest expression at 24 hpi; **E** *NtECs* peaking at 24 and 48 hpi; **F** *NtECs* with peak expression at 48 hpi. Bars represent mean relative expression levels, normalized using the mean transcript accumulation of four *N. tropicalis* endogenous genes: β-tubulin 1 (*NtβTub1*), β-tubulin 2 (*NtβTub2*), glyceraldehyde-3-phosphate dehydrogenase (*NtGAPDH*), and cytochrome c oxidase subunit III (*NtCytIII*). Error bars indicate the standard error of the mean (SEM). The primer sequences are listed in Table S5

To further characterize their expression, we performed RT-qPCR. The *NtECs* exhibited distinct expression patterns throughout the infection process and were classified into six groups based on their peak transcript accumulation (Fig. 5). Among them, *NtEC-08* showed transcript accumulation in both dormant and germinated urediniospores (Fig. 5A). *NtEC-07* displayed a pronounced expression peak in germinated urediniospores and at 24 hpi, suggesting a role during the initial penetration phase (Fig. 5B). Six effector candidates (*NtEC-02*, -*03*, *-10*, *-04*, *-11*, and *-13*) were highly expressed exclusively in germinated urediniospores, indicating potential involvement in pre-biotrophic development (Fig. 5C). *NtEC-01* and *NtEC-14* exhibited peak transcript accumulation restricted to 24 hpi, supporting their likely role in host penetration (Fig. 5D). *NtEC-05* and *NtEC-12* reached maximum expression at both 24 and 48 hpi, suggesting broader activity across early infection stages (Fig. 5E). Finally, *NtEC-09* and *NtEC-15* displayed peak transcript levels at 48 hpi, possibly reflecting a role in sustaining biotrophic interaction (Fig. 5F). To corroborate the stage-specific expression of the effector candidate genes during *N*. *tropicalis* pathogenesis, we performed a microscopic time-course analysis.

### Microscopy analyses of the compatible *V. labrusca*–*N. tropicalis* interaction

**Fig. 6:**
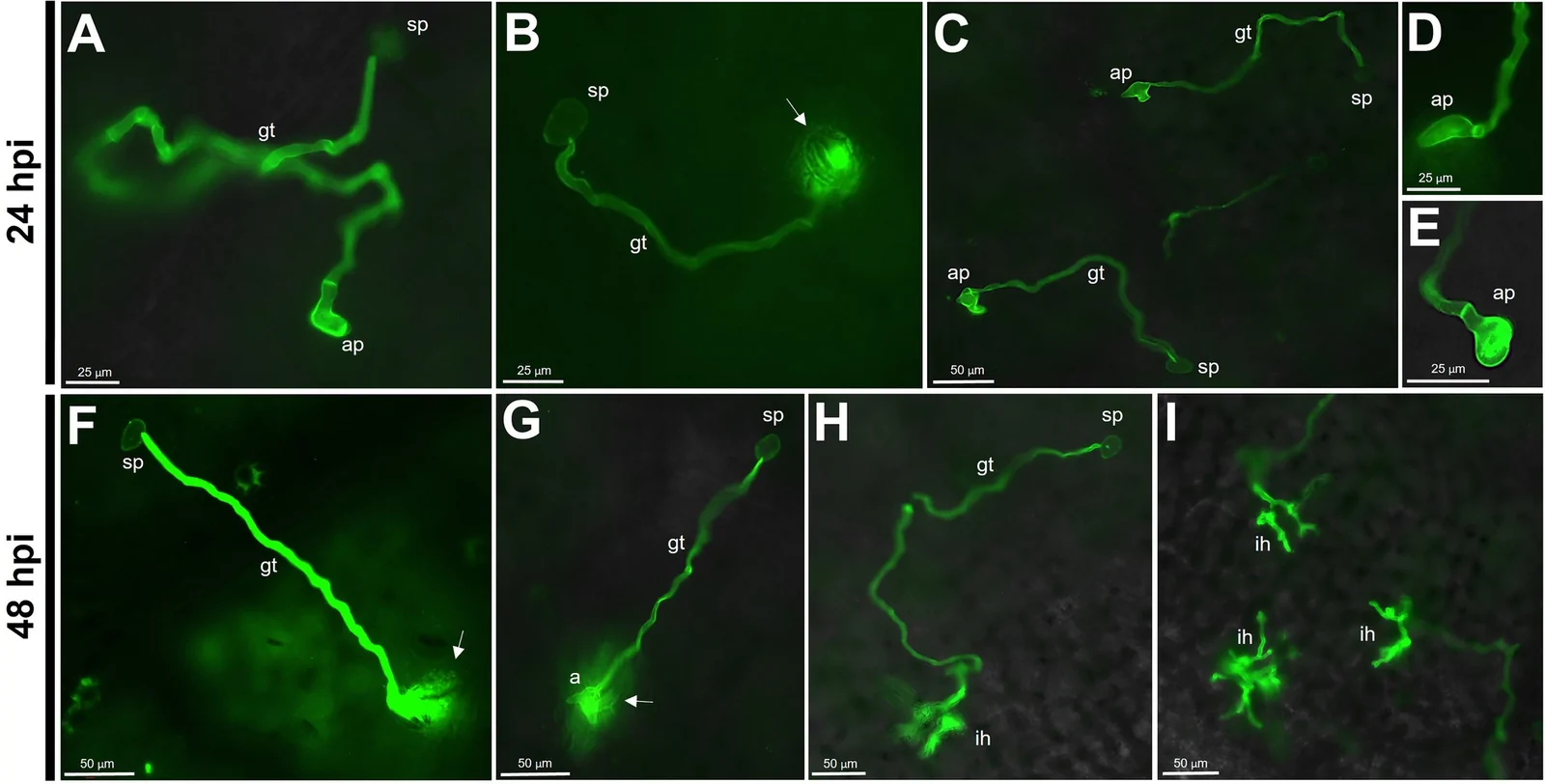
*Neophysopella tropicalis* development during early phases of infection. **A–E** Penetration phase at 24 h postinoculation (hpi). **F–I** Transition from penetration to early colonization at 48 hpi. Fully expanded leaves of *Vitis labrusca* cv. Niagara Rosada were inoculated with 1 × 10^5^ urediniospores/mL. After 24 h of incubation in a dark, humid chamber, the plants were transferred to a controlled environment with a 12-h light/12-h dark photoperiod at 25 °C. Fungal structures: urediniospores (sp), germ tubes (gt), appressoria (ap), and infection hyphae (ih) are stained green with WGA-FITC. Time points are indicated by the black lines on the left. Stomata are indicated by white arrows

To visualize the grapevine rust fungus, infected host leaf samples were stained with WGA-FITC, a lectin that binds specifically to chitin, a major component of the fungal cell wall (Marrafon-Silva et al. 2024). Microscopy analysis showed that *N. tropicalis* had reached the penetration stage at 24 hpi, as evidenced by the formation of elongated germ tube terminating in well-defined appressorium (Fig. 6A). At this time point, fungal hyphae were also observed penetrating host tissue through stomatal openings (Fig. 6B). Most germ tubes had developed appressoria at 24 hpi (Fig. 6C–E), and similar infection structures remained evident at 48 hpi (Fig. 6F, G). Intracellular infection hyphae were visible within host tissues at 48 hpi (Fig. 6H, I). Based on our experimental conditions, these findings suggest that *N*. *tropicalis* enters the early colonization stage by 48 hpi.

**Fig. 7:**
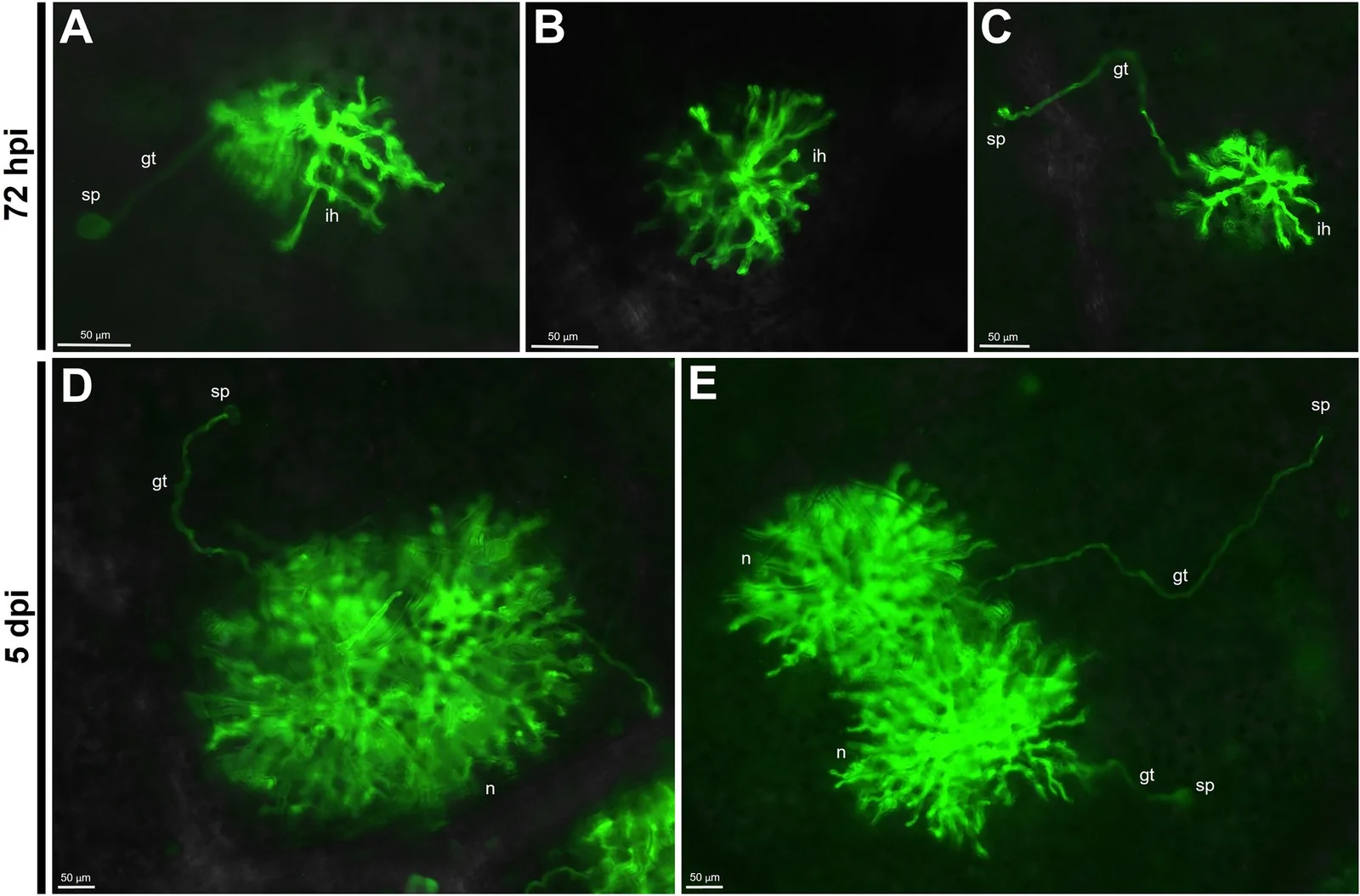
*Neophysopella tropicalis* colonization phase of infection. **A–C** Infected leaves at 72 h post-inoculation (hpi). (D–E) Infected leaves at 5 days post-inoculation (dpi). Fully expanded leaves of *Vitis labrusca* cv. Niagara Rosada were inoculated with a suspension containing 1 × 10^5^ urediniospores/mL. Following 24 h of incubation in a dark, humid chamber, the plants were transferred to a controlled environment maintained at 25 °C with a 12-h light/12-h dark photoperiod. Fungal structures, including urediniospores (sp), germ tubes (gt), appressoria (ap), infection hyphae (ih), and hyphal network (n), were visualized by staining with WGA-FITC, appearing green under fluorescence. Black lines on the left denote each time point

At 72 hpi, infection hyphae began proliferating within the grapevine leaf tissue, marking the onset of a well-established colonization stage (Fig. 7A–C). By 5 dpi, extensive radial growth of intercellular hyphae was observed, forming broad hyphal networks at the infection sites (Fig. 7D). At this point, the coalescence of hyphal networks originating from individual urediniospores is also evident (Fig. 7E). Based on these observations, the colonization phase of *N*. *tropicalis* was noticed as occurring between 72 hpi and 5 dpi.

**Fig. 8:**
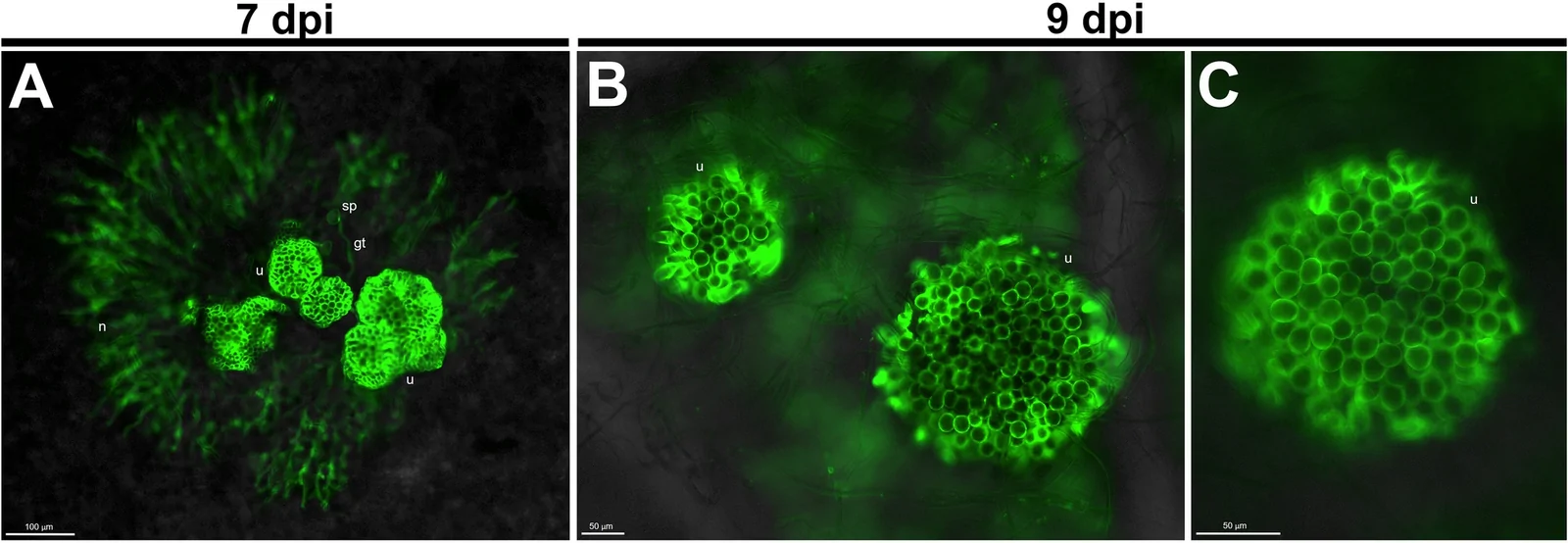
*Neophysopella tropicalis* reproductive phase. **A** Infected leaves at 7 days postinoculation (dpi). **B, C** Infected leaves at 9 dpi. Fully expanded leaves of *Vitis labrusca* cv. Niagara Rosada were inoculated with a suspension containing 1 × 10^5^ urediniospores/mL. Following 24 h of incubation in a dark, humid chamber, the plants were transferred to a controlled environment maintained at 25 °C with a 12-h light/12-h dark photoperiod. Fungal structures—including urediniospores (sp), germ tubes (gt), hyphal network (n), and uredinia (u)—were visualized by staining with WGA-FITC (green fluorescence). Time points are indicated by the black lines at the top

By 7 dpi, hyphal networks had further expanded, and both closed and partially opened uredia containing urediniospores were observed (Fig. 8A). At 9 dpi, all rust pustules exhibited fully mature, open uredia abundant in urediniospores (Fig. 8B, C), marking the reproductive phase of *N. tropicalis*. Having established a clear timeline for each infection stage, from penetration (24–48 hpi), through colonization (72 hpi to 5 dpi), and up to reproduction (7–9 dpi), we can now correlate these developmental phases with the transcript accumulation profiles of individual *NtEC* genes. The next step is to functionally characterize these candidates to identify those that act as bona fide effectors.

### *N. tropicalis* effector candidates with plant immunity-suppressing activity

To overcome the challenges of functional characterization in obligate biotrophic fungi such as rust pathogens, we employed a Type III Secretion System (T3SS)-mediated delivery method using *P. fluorescens* EtHAn. This approach enabled us to individually test the ability of NtECs to suppress plant immune response. Specifically, we tested their capacity to inhibit AvrB-triggered cell death in *N. benthamiana* leaves (Maia et al. 2022).

**Fig. 9:**
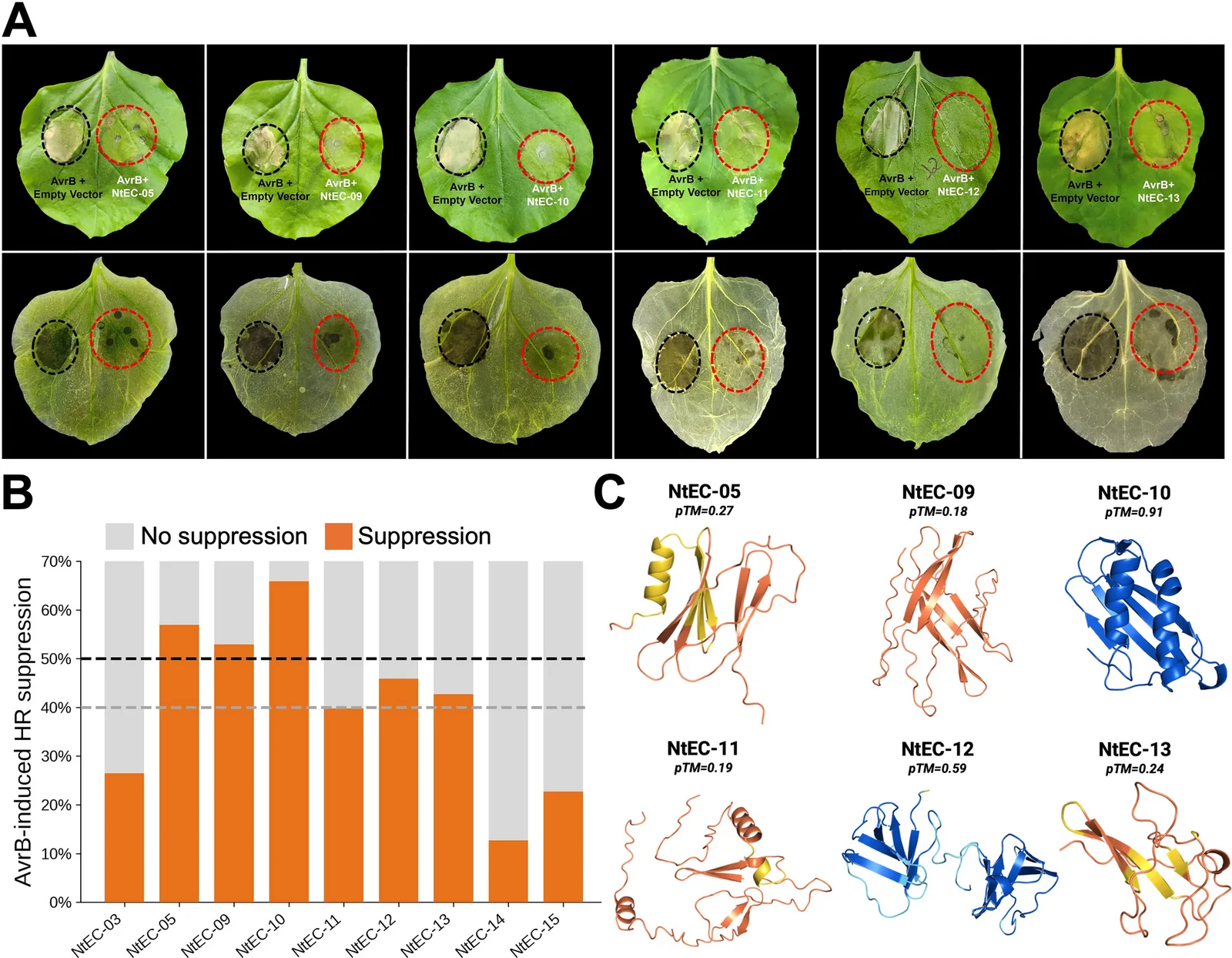
*Neophysopella tropicalis* effectors exhibiting cell death suppression activity. **A** Six effectors suppress tissue collapse associated with AvrB-induced cell death in *Nicotiana benthamiana*. Bacterial suspensions of *Pseudomonas fluorescens* EtHAn co-expressing pVSP6::*AvrB* and the empty vector pEDV6 (AvrB + empty vector) were mixed in a 1:1 ratio to a final concentration of 2 × 10⁷ CFU mL⁻^1^ and used as a negative control (black dotted circle). For experimental treatments, *P. fluorescens* EtHAn strains expressing pVSP6::*AvrB* were co-infiltrated with strains individually expressing each candidate effector (AvrB + NtEC) under the same conditions (red dotted circle). The upper panel shows leaf images captured 72 h post-infiltration, while the lower panel displays the same leaves after ethanol bleaching to enhance visualization of cell death. **B** Histogram showing the extent of AvrB-induced hypersensitive response (HR) suppression observed for nine different pEDV6::*NtEC* constructs. Suppression levels were determined based on the percentage frequency across all co-infiltrated leaves, derived from three independent experiments with ten biological replicates each (see Supplementary Table S6). Suppression levels greater than 50% (black dashed line) were categorized as moderately high, whereas levels above 40% (gray dashed line) were regarded as moderate. **C** Structural predictions of the six NtEC cell death suppressors using AlphaFold v3. Structures are color-coded based on pLDDT scores: high confidence (dark blue), medium confidence (cyan), low confidence (orange), and very low confidence (yellow). The pTM values are shown for each model

Nine *NtEC* coding sequences (*NtEC-03*, -*05*, *-09*, *-10*, *-11*, *-12*, *-13*, *-14* and *-15*), excluding their predicted native signal peptides, were cloned into the pEDV6 vector, and each pEDV6::*NtEC* construct was mobilized into *P. fluorescens* EtHAn for in planta delivery. Six of the nine candidates tested were able to suppress AvrB-induced cell death (Fig. 9A). Among these, three effectors (NtEC-05, NtEC-09, and NtEC-10) exhibited moderately high suppression activity, with consistent suppression frequencies ranging from 53 to 66% across three independent experiments (Fig. 9B; Table S6). In addition, three candidate effectors (NtEC-11, NtEC-12, and NtEC-13) showed moderate suppression activity, ranging from 40 to 46% (Fig. 9B; Table S6). The remaining candidates (NtEC-03, NtEC-14, and NtEC-15) displayed suppression levels below 30% and were therefore considered to lack cell death suppression activity in our assay (Supplementary Fig. S5).

Furthermore, the three-dimensional structures of the six cell death-suppressing NtECs were predicted using AlphaFold v3 (Abramson et al. 2024). Low-confidence predictions (pTM < 0.5) were obtained for NtEC-05, NtEC-09, NtEC-11, and NtEC-13 (Fig. 9C), while NtEC-10 and NtEC-12 yielded high-confidence models (pTM > 0.5). Notably, NtEC-10 exhibited both high structural similarity and significant sequence identity with a predicted glycan-binding protein Y3-like domain-containing protein from *Phakopsora pachyrhizi* (UniProt ID: A0A0S1MKU5) (Supplementary Fig. S6). Together, these results highlight a subset of *N. tropicalis* effectors with potential roles in host immunity suppression and offer initial structural insights to guide future functional studies.

## Discussion

To our knowledge, this study provides the first transcriptomic analysis of *N. tropicalis*. We focused on the urediniospore germination stage because it represents a key moment when the fungus initiates the infection process (Shen et al. 2017; Lorrain et al. 2019). By examining this stage, we identified genes that represent strong candidates for encoding effector proteins likely important in preparing the pathogen for host penetration, suppressing early immune responses, and establishing a compatible interaction. From the more than 64,000 assembled transcripts, we predicted 303 genes encoding secreted proteins with features typical of fungal effectors. This dataset considerably expands our understanding of the *N. tropicalis* secretome and provides a collection of high-confidence effector candidates suitable for functional assays to identify bona fide effectors with immune-suppressing activity and broader relevance to grapevine–rust interactions. Early transcriptional mobilization and protein secretion are also observed in other biotrophic rust pathogens, such as *Puccinia basss* f. sp. *tritici* and *Puccinia triticina* (Tao et al. 2026; Ozketen et al. 2026), where researchers identified an early investment in candidate effector secretion, alongside other pathogenicity-related proteins, to prepare the infection pathway. As such, our comparative data support the model that obligate rust fungi proactively deploy their molecular secretome during pre-infection phases to successfully prime the host environment.

Beyond the identification of candidate effector genes, our transcriptome assembly revealed full-length ORFs for a second β-tubulin gene (*NtβTub2*), not previously reported in *N. tropicalis*, as well as for a glyceraldehyde-3-phosphate dehydrogenase (*NtGAPDH*) gene. The availability of the complete β-tubulin sequence is particularly relevant because this gene is a well-established target of benzimidazole fungicides, and its full coding sequence facilitates the monitoring of the canonical mutations associated with fungicide resistance (Zhou et al. 2016). In addition, the complete coding sequences of both *β-tubulin* and *GAPDH* also may serve as reliable endogenous reference genes, making them useful for accurate normalization in RT-qPCR expression studies. These genes therefore represent valuable molecular resources that will support future genetic, epidemiological, and population studies of this economically important rust fungus.

Analysis of the predicted candidate effectors revealed a remarkable diversity within the *N. tropicalis* effector repertoire. Sequence clustering grouped the 303 predicted candidate effectors into 142 sequence families, with nearly half of these families represented by a single member. This predominance of singleton and small families indicates that the *N. tropicalis* secretome is composed primarily of highly divergent proteins rather than a few expanded paralogous gene families. A similar pattern has been reported in other rust fungi, where sequence-based clustering analyses revealed that secreted proteins are frequently organized into numerous lineage-specific families with limited sequence conservation (Saunders et al. 2012). Such diversification is thought to reflect the intense evolutionary pressure imposed by host immune systems, which favors the rapid evolution of effector sequences while maintaining their ability to manipulate conserved host cellular processes (Lorrain et al. 2019; Figueroa et al. 2021).

The clustering analysis also provides important biological context for selecting the fifteen most highly expressed candidate effectors. Although these *NtECs* were among the most abundant transcripts during urediniospore germination, they represented thirteen distinct sequence families, indicating that the early transcriptional program of *N. tropicalis* is distributed across diverse effector lineages rather than concentrated within a few expanded gene families. Furthermore, by initiating this complex program prior to host penetration, *N. tropicalis* is capable of presenting this highly diversified array of initial signals, likely optimizing its stealth and capability to neutralize early plant immune responses (Osherov and May 2000; Duplessis et al. 2011). To gain further insight into the potential roles of these candidate effectors, we analyzed their expression throughout different stages of pathogenesis using RT-qPCR. The results revealed distinct temporal expression profiles, supporting functional specialization within the *N*. *tropicalis* effector repertoire. This stage-specific expression is consistent with the sequential deployment of effector genes reported in other filamentous pathogens (Kleemann et al. 2012; Jaswal et al. 2020).

Eight *NtECs* (e.g., *NtEC*-*02*, -*03*, -*04*, -*07*, -*08*, -*10*, -*11*, -*13*) were preferentially expressed in germinated urediniospores, pointing to roles in pre-biotrophic development and preparation for host entry. Others, such as *NtEC-01* and *NtEC-14*, showed maximum expression exclusively at 24 hpi, consistent with a function in penetration and appressorium-associated processes. *NtEC-05* and *NtEC-12* displayed sustained expression across early stages, suggesting functions that extend from penetration through the initial establishment of biotrophy. Finally, *NtEC-09* and *NtEC-15* reached maximal expression at 48 hpi, indicating a role in sustaining early colonization. To further support these temporal expression profiles within the biological context of infection, we coupled RT-qPCR analyses with histological observations using WGA-FITC staining (Marrafon-Silva et al. (2024)).

At 24 hpi, *N. tropicalis* had developed fully differentiated appressoria with clear septation and initiated host penetration, corresponding tightly with the peak expression of five *NtEC* genes (*NtEC-01*, *-05*, *-07*, *-12*, *-14*). The close timing between appressorium formation and candidate effector induction suggests that these proteins are released just before or during host penetration when the fungus first encounters plant immune surveillance. Appressoria were frequently positioned directly above stomatal openings, indicating that *N*. *tropicalis* may exploit these natural openings in grapevine leaves as entry points into host tissues. Stomata, however, are not merely passive entry sites but function as dynamic immune structures capable of perceiving microbial signals and activating defense responses (Melotto et al. 2024). Guard cells recognize fungal cell-wall-derived chitin via the CERK1 receptor complex, triggering calcium influx, activating S-type anion channels, and rapid stomatal closure to restrict pathogen ingress (Ye et al. 2020). To circumvent this, some fungi bypass stomatal immunity by converting chitin to chitosan oligosaccharides or secreting effectors that interfere with guard-cell signaling, thereby preventing closure or inducing guard-cell death. The high expression of these five *NtECs* during penetration suggests that they may contribute to suppressing guard-cell-mediated defenses or altering stomatal signaling pathways, facilitating *N*. *tropicalis* entry through these openings. By interfering with stomatal immunity at this early stage, *N*. *tropicalis* likely achieves efficient penetration while minimizing host recognition and the energetic cost of direct cuticle degradation. Following successful entry, infection progressed rapidly. By 48 hpi, small infection hyphae were visible, marking the transition from penetration to early colonization and coinciding with the maximal expression of *NtEC-09* and *NtEC-15*, which may contribute to the establishment and maintenance of early biotrophic growth. All these stage-specific expressions are consistent with the sequential deployment of effector genes in successive waves, a well-documented hallmark of obligate biotrophic rust pathogens (Duplessis et al. 2014; Lorrain et al. 2019).

Although none of the *NtECs* showed preferential expression at later stages of infection, WGA-FITC staining revealed that between 72 hpi and 5 dpi, *N*. *tropicalis* entered an active colonization phase characterized by extensive proliferation of infection hyphae within host tissues. By 7–9 dpi, fungal biomass was widespread, with expanding hyphal networks giving rise to rust pustules containing mature uredia. Together, these histological observations offer an unprecedented timeline of the *N. tropicalis–V. labrusca* compatible interaction and establish a clear framework for interpreting candidate effector gene expression in the context of fungal development.

A major advance of this study was the functional analysis of NtEC proteins using the *P*. *fluorescens* EtHAn-adapted effector detector vector (EDV) system to evaluate their ability to suppress AvrB-triggered effector-triggered immunity (ETI) in *N*. *benthamiana* (Maia et al. 2022). Six effectors (NtEC-05, -09, -10, -11, -12, and -13) consistently suppressed ETI, with NtEC-05, NtEC-09, and NtEC-10 exhibiting the strongest activity. Interestingly, while NtEC-05 appears to be specific to the order Pucciniales and NtEC-10 is broadly conserved across Basidiomycota and Ascomycota, NtEC-09 has no homologs in current databases, suggesting it represents a pioneer effector unique to *N. tropicalis*. These ETI-suppressing effectors represented multiple independent sequence families identified by the clustering analysis. Analogous AvrB-triggered ETI suppression has also been documented in the coffee rust fungus *Hemileia vastatrix*, where five effector candidates (HvEC_006, HvEC_032, HvEC_043, HvEC_053 and HvEC_081) attenuated AvrB-triggered HR, with HvEC_043 exhibiting the strongest suppressive activity (Maia et al. 2026). Similarly, suppression of AvrB-triggered ETI has been reported for effectors from the evolutionarily distant smut fungus *Sporisorium scitamineum* (Teixeira-Silva et al. 2020; Maia et al. 2022). Together, these observations suggest that unrelated fungal pathogens may have independently evolved effectors that interfere with conserved host immune pathways associated with AvrB-triggered defense, consistent with evolutionary convergence in plant–pathogen interactions (Mukhtar et al. 2011).

The molecular basis of AvrB-triggered ETI provides a mechanistic framework for interpreting how these fungal effectors may suppress host immunity. Following its delivery into the plant cell, the bacterial effector AvrB targets RPM1-INTERACTING PROTEIN 4 (RIN4), a plasma membrane-associated regulator that integrates PTI (pattern-triggered immunity) and ETI. RIN4 is now recognized as a central hub of plant immune signaling and convergence point for multiple bacterial virulence effectors. A number of *Pseudomonas syringae* effectors (AvrB, AvrRpm1, AvrRpt2, AvrPto, AvrPtoB, and HopF2) are known to target RIN4 or its associated signaling network through distinct molecular mechanisms to dampen plant immune responses, highlighting this protein as a central hub in plant–pathogen interactions (Ray et al. 2019; Mackey et al. 2003). Biochemical evidence has shown that AvrB is a glycosyltransferase that modifies RIN4 directly by rhamnosylation, thereby establishing a direct link between enzyme activity and immune modulation (Peng et al. 2024). Most recently, Lee et al. (2026) demonstrated that AvrB-triggered modifications of RIN4 block its interaction with the receptor-like cytoplasmic kinase BIK1, thereby suppressing PTI and activating RPM1-mediated ETI.

In this scenario, the ability of NtECs to suppress AvrB-triggered ETI implies that these fungal effectors likely interfere with host immune signaling events that occur after the perception of AvrB, rather than directly targeting the bacterial effector. Whether *N. tropicalis* effectors also target RIN4 or interfere with other conserved immune regulators remains an important question for future investigation. More recently, structural studies demonstrated that the *Phytophthora infestans* effector AVRcap1b inhibits ETI by binding to activated helper NRC3 proteins and preventing the assembly of the resistosome, revealing that pathogen effectors can interfere directly with the execution of immunity and not just with upstream signaling events (Seager et al. 2026). The identification of virulence targets of the NtECs will be essential for understanding the molecular mechanisms by which *N. tropicalis* suppresses host immunity and establishes compatibility with grapevine.

Structural predictions generated by AlphaFold 3 provide preliminary clues into *N. tropicalis* effector architecture. While NtEC-05, NtEC-09, NtEC-11, and NtEC-13 produced low-confidence structural models, NtEC-10 and NtEC-12 yielded well-defined 3D predictions. Notably, NtEC-10 exhibits structural similarity to a predicted glycan-binding protein domain from *P. pachyrhizi*, suggesting a conserved biochemical role potentially related to host cell wall interactions (Tanaka and Kahmann 2021). These predicted folds also offer a basis for comparative analyses with effectors from other rust fungi, where structural conservation often persists despite limited sequence similarity, thereby revealing conserved molecular functions and immune suppression mechanisms (Le Naour-Vernet et al. 2025).

Overall, this work provides the first comprehensive characterization of candidate effectors from *N*. *tropicalis*, establishing a foundation for future studies of grapevine–rust interactions. The *N. tropicalis* effectors identified here could serve as molecular probes to screen grapevine germplasm for recognition-based resistance, ultimately supporting the development of effector-informed breeding strategies, as demonstrated for other viticultural diseases (Paineau et al. 2025). Cross-species comparisons of effector repertoires may further reveal conserved virulence determinants that could serve as durable resistance targets. Subsequent studies should focus on: (i) identifying the host targets of NtECs to elucidate their mechanisms of immune suppression, (ii) validating their functions directly in grapevine through transient expression assays, and (iii) expanding genomic resources for *N. tropicalis* to better contextualize effector evolution. Collectively, these efforts will deepen our understanding of the molecular mechanisms underlying Asian grapevine leaf rust and accelerate the development of durable resistance strategies.

## Supplementary Information

Below is the link to the electronic supplementary material.

Supplementary file1 (JPG 153 KB)

Supplementary file2 (JPG 114 KB)

Supplementary file3 (JPG 1300 KB)

Supplementary file4 (JPG 201 KB)

Supplementary file5 (JPG 1272 KB)

Supplementary file6 (JPG 168 KB)

Supplementary file7 (XLSX 28 KB)

## Data Availability

No datasets were generated or analyzed during the current study.
